# Developments in Ureteral Stent Technology

**DOI:** 10.3389/fsurg.2021.764167

**Published:** 2021-11-17

**Authors:** Justin Lee, Matthew Katz, Ojas Shah

**Affiliations:** Department of Urology, Columbia University Medical Center, New York, NY, United States

**Keywords:** ureteral stent, silicone, ureteroscopy, urolithiasis, kidney stones

## Abstract

Ureteral stents have been utilized for decades in maintaining ureteral patency, most commonly after ureteroscopy in the treatment of urolithiasis. Since their initial development, ureteral stents have had many technological advances that have allowed for better patient outcomes with improvements in comfort, durability, patency, encrustation resistance, biocompatibility, ease of insertion, migration, and biofilm development. Several new ureteral stents enter the market every year, each with their own touted benefits. It is essential to understand the different advantages for each ureteral stent to provide the best available care to patients when possible. The purpose of this review is to give a brief history of ureteral stent development and summarize the recent developments in ureteral stent designs. We aim to review the data supporting the clinical advantages of the latest ureteral stents available for use by urologists.

## Introduction

Ureteral pigtail stents were first introduced in 1978 and have been in use for decades to maintain ureteral patency (1). They are frequently used peri-operatively in the management of urolithiasis, as well as other ureteral conditions, to prevent or treat ureteral obstruction. While stents have known side effects including patient discomfort, biofilm and encrustation, urologists continue to use them routinely after upper urinary tract endoscopy to relieve and/or prevent obstruction and to cause passive dilation of the ureter. Given the frequent use of stents in endourologic practice, it is important to understand the properties of the various stents available with respect to patient discomfort, biocompatibility, migration, ease of insertion, encrustation, and biofilm development. There has been a tremendous amount of work in this space to try and create the “ideal” stent, however the holy grail of stents has still not been developed. Since the introduction of ureteral stents, several different kinds have been developed with different compositions, coatings and designs which all have different clinical impacts.

Silicone was used as the initial polymer for ureteral stents when they were first introduced. Several advantages of silicone material have been reported including its soft composition, biocompatibility (2) and lower encrustation rates (3) than other stent materials. Despite these advantages, silicone stents were previously deemed impractical due to both the high frictional coefficients making placement difficult and lower tensile strength (2). More recently, advances in technology have allowed for modern silicone stents to be developed with stronger tensile strengths, but still with softer compositions, potentially allowing for less stent-related discomfort (4). Coating of the silicone stents with hydrophilic material has allowed for easier stent placement (5). For these reasons, modern silicone stents have emerged as the latest ureteral stents which come with several significant advantages. Other new stents introduced recently include the pigtail suture stent (PSS) where the distal portion of the stent is a 0.3 Fr suture that terminates in the bladder and the Tria™ stent which has a hydrophobic coating to decrease encrustation and biofilm.

The goal of this review is to discuss the latest developments in ureteral stent technology and their potential roles in clinical urologic practice.

## Tria™ Stents

One of the new ureteral stents developed is the Tria™ stent with Percushield™ coating (Boston Scientific). The main advantage of this stent design is a novel nonionic, smooth, hydrophobic inner and outer Percushield™ coating that claims to reduce adhesion with calcium and magnesium salts to prevent stent encrustation. The initial study was done on an *in vitro* model with the Tria™ stents (*n* = 15) incubated in sterile urine baths and in *Proteus mirabilis* bacterial infection urine baths for 2 weeks. There was reportedly significantly less deposition of calcium and magnesium salts in both sterile and *Proteus* urine baths compared to competitor stents (*n* = 15) (Table 1) (Boston Scientific 2021). Currently, there is a lack of studies directly comparing rates of ureteral stent encrustation among various stents (6). A recent study (*n* = 84) compared the 14-day encrustation rates between Tria Ureteral Stents with Percushield™ to the Polaris Ultra ureteral stents with HydroPlus coating™ (Boston Scientific). Using micro-CT to measure encrustation rates, the Tria™ and Polaris Ultra™ stents had comparable inner encrustation volume (*p* = 0.183) and had similar outer/total surface encrustation volumes at 14 days. Interestingly, this is the first study to employ a micro-CT method in analysis of ureteral stent encrustation. One limitation of this study is the short follow-up period of 14 days as encrustation becomes more of a concern with longer dwell times. There are no other published studies examining the rates of encrustation with Tria™ stents *in vivo*. The benefit of the Tria™ stent is that it is similar to other polyurethane stents, so it is very easy to place. It also comes in two different tensile strengths as either a firm or soft stent. The firm stents are theoretically better for obstruction from extrinsic compression vs. the softer stent is theoretically more comfortable for the patient. Further long-term data is needed to determine the efficacy of the Tria™ stents in preventing encrustation compared to other ureteral stents.

**Table 1:** Differences in rates of combined calcium and magnesium encrustation for Tria™ Soft (Boston Scientific) ureteral stents compared to competitor stents at 2 weeks.

	**Bard Inlay Optima™**	**Coloplast Imajin™**	**Cook Black Silicone™**
Sterile urine bath	−60%	−52%	−48%
Proteus urine bath	−19%	−45%	−14%

*All changes were reported to be statistically significant, but p-values were not provided (Boston Scientific website 2021)*.

## Pigtail Suture Stent

More recently, a newer type of ureteral stent was developed to help with ureteral stent symptoms called a pigtail suture stent (PSS) where the distal portion of the stent is a 0.3 Fr suture that terminates in the bladder (Jfil™ stent, Rocamed). The concept of this design was derived from the theory that the distal curl in the bladder is related to stent colic, stent reflux, and irritative lower urinary tract symptoms. A prospective cohort study with 78 patients was done with JFil™ vs. a conventional hydrophilic double pigtail stent (Vortek™, Coloplast) following flexible ureteroscopy for stone treatment (7). Stents were removed 2 weeks after surgery. Ureteral stent symptom questionnaire (USSQ) pain scores were done 2 days, 2 weeks, and 6 weeks after surgery. Urinary Symptom Index USSQ scores were significantly lower in the PSS group at 2 weeks (*p* = 0.022) and 2 days (*p* = 0.001). Additionally, overall visual analog scale pain scores (*p* = 0.002), body pain scores (*p* = 0.021), and general health index score (*p* = 0.036) were significantly better in the Jfil™ group compared to the double pigtail stent group. After the 2-week scores were adjusted for baseline scores (6 week after surgery), the above scores remained statistically significant in favor of the Jfil™ group. Importantly, the patients in the Jfil™ group reported significantly lower scores for urinary frequency, sensation of incomplete emptying, and burning while voiding. There were no cases of stent dislodgement or worsening hydronephrosis reported in either group, suggesting that the Jfil™ stent was effective in preventing ureteral obstruction, despite having a suture for the distal portion of the stent.

There are two key limitations with the Bosio et al. study. One is that they used polyurethane stents as the direct comparison, while it is known that polyurethane stents are not necessarily the best stents for preventing stent related symptoms. Additionally, as pointed out by Ventimiglia et al. in a letter to the editor, the authors placed Jfil™ stents in patients after uncomplicated ureteroscopy, when in fact current EAU guidelines recommend no ureteral stent placement after uncomplicated ureteroscopy (although still commonly performed globally).

Another randomized controlled study found significantly lower pain scores and analgesic requirements in a PSS group compared to a conventional polyurethane double pigtail stent after uncomplicated URS for symptomatic ureteral stones (8). This new type of ureteral stent may be effective following flexible ureteroscopy while also reducing stent-related symptoms compared to other ureteral stents (9). They also reported clear ureteral dilation in all 28 of the patients who had PSS placed after a 1 month indwelling period. There has been one other study to date examining the effects of PSS on passive ureteral dilation. Majdalny et al. (10) placed PSS in pig ureters and found on POD13-15 that there was ureteral dilation in 5 of 6 ureters stented with PSS. This passive ureteral dilation may help facilitate access prior to ureteroscopy or postoperatively to facilitate stone fragment/dust passage after the stent is removed. Further studies are needed to definitively state whether pigtail suture stents can safely be used after ureteroscopy and if they lead to less stent related symptoms when compared to the most comfortable stents currently available.

## Biodegradable Stents

One of the recent developments in ureteral stent technology is the biodegradable stent, which is designed to dissolve in urine over time. These stents have wide-ranging advantages such as avoiding a second operation or procedure to remove stents and to avoid the feared complications of a forgotten stent. Many different biodegradable stents have been developed over the past few decades with the first reported in 2002 and 2003 (11, 12), but none have been able to show consistent degradation without complication over time *in vivo*. More recently, a group in Portugal has developed the HydrUStent™, which is made from an aqueous solution of gelatin-alginic-acid sodium salt and bismuth carbonate basic. These stents have only been tested in porcine models, although they promisingly showed all stents dissolved after 10 days (13). Another model used glycomer 631 and pure polyglycolic acid in a novel stent that dissolved completely after 3–6 weeks in porcine models without any complications at 5-month follow-up (14). This year, a new stent made from biodegradable polyurethane, magnesium, and calcium were showed to dissolve completely after 4 weeks in a porcine model (15). The clinical studies surrounding these types of stents is very limited with further need for human studies before utilization of these stents can be justified. However, this is a promising technology which may have several advantageous implications in the future.

## Modern Silicone Stents

Modern silicone stents have been developed with properties like polyurethane stents which increase ease of placement but still with softer compositions and improved biocompatibility compared to polyurethane. The softer compositions are thought to lead to better symptomatic outcomes for patients (16). Given the recent emergence of these modern silicone stents, there is a paucity of data surrounding the clinical impact of these stents on patient outcomes compared to other stents. Wiseman and associates performed a single-blinded, randomized multicenter study examined the effects of quality of life after placement of hydrocoated silicone ureteral stents (Imajin Hydro™, Coloplast) vs. hydrocoated nonsilicone ureteral stents (Percuflex Plus™, Boston Scientific) after flexible ureteroscopy (17). In the group of 141 eligible, randomly assigned patients, the silicone group had significantly lower USSQ scores compared to the nonsilicone group (18.7 vs. 25.1, *p* = 0.015) at postoperative day 20 (POD20) (Table 2). After normalizing the pain scores to consider differences in score reports between men and women, the differences remained significant (*p* = 0.013). Other urinary symptom scores were also significantly lower in the silicone group at POD20 (26 vs. 31). Safety outcomes were similar between the two stent groups. Although it was a relatively small study, the results suggest the modern silicone Imajin Hydro™ stent offers a safe option with lower pain and urinary symptom scores following flexible ureteroscopy when compared to nonsilicone Percuflex Plus™ stents. One of the limitations of this study is that a standard 26 cm length was used in all patients, which may affect stent-related pain for patients with different heights or ureteral lengths. A criticism of the study was that stents were left in patients for several weeks, where the benefit was most significant, as opposed to the typical dwell times of 5–10 days following stone treatment. Similarly, Gadzhiev et al. performed a small randomized study which found that silicone stents (Black Filiform™, Cook Medical) were associated with lower visual analog scale pain scale scores at 2 weeks (*p* = 0.023) and prior to stent removal at 4 weeks (*p* = 0.001) compared to polyurethane stents (Rüsch, Teleflex) (Table 2) (4).

**Table 2:** Silicone ureteral stents vs. non-silicone ureteral stents in pain scores and biofilm/encrustation formation (4, 17, 18).

**Author**		**Silicone stents**	**Non-silicone stents**	***p***
Wiseman et al. (17)		**Imajin Hydro**™ **(Coloplast)**	**Percuflex Plus**™ **(Boston Scientific)**	
	USSQ body pain scores @ Day 20	18.7 (11.4)	25.1 (14.2)	0.015
	Gender normalized scores	19.2 (11.9)	26.0 (15.1)	0.013
Gadzhiev et al. (4)		**Black Filiform**™ **(Cook Medical)**	**Polyurethane (Rüsch, Teleflex)**	
	Visual analog pain scores @ Day 14	1.1	2.4	0.0223
Barghouthy et al. (18)		**Imajin Hydro**™ **(Coloplast)**	**Percuflex Plus**™ **(Boston Scientific)**	
	Rate of surface biofilm (global) @ Day 20	0.93 (0.09)	1.24 (0.08)	0.0021
	Rate of surface encrustation (global) @ Day 20	0.78 ± 0.11	1.22 (0.10)	0.0048

Another essential aspect of stent practicality is the rate of biofilm formation and stent encrustation since they are common causes of stent obstruction and infection (19) sometimes requiring complex interventions to change or remove. Barghouthy et al. (18) underwent complex analysis of the Imajin Hydro™ (Coloplast) and Percuflex Plus™ (Boston Scientific) stents that were removed on POD20 for formation and encrustation. The rates of biofilm formation on the internal and external parts of the stent were 25% lower (*p* = 0.002) in the silicone stents. The rate of encrustation was 36% lower in the silicone group (*p* = 0.004) as well (Table 2). Only in the ureteral shaft portion of the stent did the two types of stents have similar rates of encrustation and biofilm formation. This study helped clearly demonstrate the advantage of silicone stents over non-silicone stents with respect to encrustation and biofilm formation rates. Interestingly, these significant differences did not translate into different rates of UTIs since they were similar in both groups (5). In another study, silicone stents were found to have significantly lower rates of encrustation and biofilm development in stone formers compared to polyurethane stents (20). In an *in vitro* model with 5 different types of stents soaked in artificial urine for 14 weeks, silicone stents were found to have significantly lower rates of encrustation with struvite and hydroxyapatite compared to all other stents (Polyurethane, HPU, Percuflex, Silitek) (21). This effect is thought to be due to the uniform surface. Based on the current literature, silicone stents have been associated with lower rates of encrustation and biofilm development than other stent types.

Historically silicone stents were abandoned due to the difficulty in placing the stent over a guidewire. The modern silicone stents with hydrophilic coatings are now able to be placed over a guidewire. When deploying a 6 Fr silicone stent, it is best to use a hydrophilic guidewire to enable placement, as the smaller lumen will not easily pass over PTFE-coated (polytetrafluoroethylene) or hybrid (PTFE-Nitinol) guidewires. In contrast, the 7 Fr and 8 Fr stents can be placed over a standard hybrid guidewire, but still easiest when placed over a hydrophilic guidewire. In our experience, when placing a silicone stent, it is helpful to submerge the stent in saline to optimize the hydrophilic coating and apply a small amount of lubrication to the stent and/or wire to ease the placement when using a non-hydrophilic guidewire.

## How to Choose the Right Stent

The ideal stent is one that causes minimal to no discomfort, causes minimal urinary symptoms, has no encrustation or biofilm formation, is easy to insert, and is radiopaque. While there is currently no stent on the market that is perfect, the modern silicone stent seems to provide some of the properties of the ideal stent based on the available research. The additional newer stents require more clinical investigation but may prove to be just as useful in a urologist's armamentarium. Table 3 shows our recommended types of newer ureteral stents based on various indications for ureteral stenting. The times in which silicone are less ideal is in cases of extrinsic compression causing obstruction or difficult strictures due to the decreased tensile strength of silicone compared to other stents. In those cases, polyurethane stents, stents with wire-reinforcement, or possibly metallic stents are better suited. In general, stents are uncomfortable and as urologists we should use strategies to optimize patient comfort and decrease the costs and decreased quality of life associated with stent bother. This includes minimizing stent dwell time, use of adjunct medication to help with stent colic, and going stent-free when safe. Patient education and setting expectations also remains a crucial component in preventing unnecessary costs from stent bother. Further research into stent technology is needed to continue to optimize the patient experience and to create the ideal stent.

**Table 3:** Recommendations for different novel ureteral stent options based on various indications.

**Indication**	Newer stent options^*^	**Reasoning**
Stenting after ureteroscopy	Coloplast Imajin Hydro™ Cook Black™	Silicone stents offer softer compositions and potentially lower stent related symptoms, lower rates of encrustation and biofilm
	Tria Soft™	Tria Soft™ likely more comfortable than the Tria Firm™ with decreased risk of encrustation
	J-fil™	Decreased stent reflux and irritative lower urinary tract symptoms
Obstructing ureteral and/or renal stone	Coloplast Imajin Hydro™ Cook Black™	Increased comfort, decreased risk of encrustation/biofilm for longer dwell times
Pyonephrosis	Tria Soft™	
Malignant ureteral obstruction	Tria Firm™	Longer lasting with reported lower risk of encrustation. Avoid silicone as it is not rigid enough to resist severe extrinsic obstruction
Ureteral stricture	Coloplast Imajin Hydro™	Longer lasting with reportedly lower encrustation rates; potential for increased comfort
	Cook Black™	
	Tria Soft™	
	Tria Firm™	

* *There are many current stent options that will also function in these clinical scenarios, however the focus of this table are on the newer stent technologies specifically discussed in this article*.

## Author Contributions

JL and MK contributed to literature search and writing. OS contributed to conception, literature search, and writing. All authors contributed to the article and approved the submitted version.

## Conflict of Interest

MK: consultant for Boston Scientific. OS: advisory board and lecturer for Boston Scientific, advisory board and lecturer for Coloplast. The remaining author declares that the research was conducted in the absence of any commercial or financial relationships that could be construed as a potential conflict of interest.

## Publisher's Note

All claims expressed in this article are solely those of the authors and do not necessarily represent those of their affiliated organizations, or those of the publisher, the editors and the reviewers. Any product that may be evaluated in this article, or claim that may be made by its manufacturer, is not guaranteed or endorsed by the publisher.
